# Revealing the Diversity of the Mycobiome in Different Phases of Ticks: ITS Gene-Based Analysis

**DOI:** 10.1155/2024/8814592

**Published:** 2024-01-08

**Authors:** Shiwei Sun, Yulian Lin, Jing Han, Zhen He, Lin Zhang, Qi Zhou, Ruishan Li, Wenkai Zhang, Zhenhua Lu, Zhongjun Shao

**Affiliations:** ^1^Department of Epidemiology, School of Public Health, Air Force Medical University, Xi'an 710032, China; ^2^Baotou Medical College, Baotou 014040, China; ^3^Gansu University of Chinese Medicine, Lanzhou 730000, China; ^4^Shanxi University of Chinese Medicine, Xianyang 712046, China

## Abstract

Ticks are obligate ectoparasites and vectors of a variety of pathogens in humans and animals. Certain tick-borne pathogens (TBPs) have been identified as the cause of zoonoses, posing potentially significant threats to the human health and livestock industries. Fungi are one of the major TBPs that can affect ticks and cause disease in humans. At present, there are few studies on the diversity of fungal microbial communities carried by *Ixodes*. Therefore, profiling tick-borne fungi will contribute to understanding the tick-fungal interaction. This study evaluated the community profile and differences in the fungal microbiome in *Ixodidae* collected on parasitic ticks or nonparasitic ticks in Wuwei, Gansu Province, China. The Shannon index, Simpson index, and Richness index were used to evaluate the diversity of mycobiome. Principle coordinates analysis (PCoA) was conducted to determine patterns of diversity in mycobiome. Using correlation analysis to determine the correlation of mycobiome. The results show that the high-throughput sequencing of the internal transcribed spacer gene generated 3,634,943 raw reads and 7,482 amplicon sequence variants. The dominant tick species in this region was *Dermacentor nuttalli* (Ixodidae). The mycobiome belonged to four classes—*Dothideomycetes*, *Sordariomycetes*, *Ustilaginomycetes*, and *Tremellomycetes*—and more than 261 genera, the most abundant genera were *Cladosporium*, *Purpureocillium*, *Aureobasidium*, *Tranzscheliella*, and *Sporormiella*. Alpha diversity indicated that the abundance and evenness of mycobiome were marginally higher in nonparasitic ticks than in parasitic ticks. PCoA showed that the community structures of parasitic ticks vary from nonparasitic ticks, samples from nonparasitic ticks tended to cluster more closely than those from the parasitic ticks. Correlation analysis indicated that there was a significant positive correlation or negative correlation between the mycobiome. Our results indicate that the mycobiome carried by *Dermacentor nuttalli* had rich diversity, and there was a significant difference in mycobiome between parasitic ticks and nonparasitic ticks. These findings may conducive to understand the complex interaction between ticks and commensal fungi and provide help for the further exploration of the behavioral characteristics of ticks and formulation of effective biological control measures.

## 1. Introduction

Ticks are hematophagous ectoparasites, with approximately 900 known species worldwide [1]. As the second largest pathogen vector in the world after mosquitoes, ticks harbor, and transmit several pathogens during hematophagy [2, 3], such as tick-borne encephalitis; the spotted fever group rickettsiae; and *Anaplasma*, *Coxiella*, *Ehrlichia*, and *Babesia* spp. Environmental degradation and climate change have favored the emergence of tick-borne diseases [4, 5]. According to reports, in many countries around the world, tick-borne diseases cause incalculable economic losses and have a great negative impact on animal husbandry every year [6–8]. Ticks belong to three families: Argasidae, Ixodidae, and Nuttalliellidae [9, 10]. Approximately 177 species from the genera *Argas*, *Carios*, *Ornithodoros*, *Amblyomma*, *Anomalohimalaya*, *Dermacentor*, *Haemaphysalis*, *Hyalomma*, *Ixodes*, and *Rhipicephalus* have been identified in China [11, 12]. *Dermacentor nuttalli* is commonly found in grasslands in northern China [13] and transmits zoonotic diseases, posing a significant threat to humans and animals [2, 14].

In recent years, an increasing number of studies have demonstrated that the microbiota of ticks plays an indispensable role in the vector capacity and pathogen transmission kinetics of many tick-transmitted diseases [15–17]. For example, a previous study showed that the endosymbiotic bacteria of ticks not only plays a key role in reproductive health and nutrient provision but also influence pathogen acquisition, virulence, and transmission [17–19]. However, little is known about the diversity of the mycobiome in ticks, fungus/insect interactions are well-characterized [20]. Entomopathogenic fungi are represented in five fungal taxa, and insect hosts are represented in 20 insect orders [21]. This makes entomopathogenic fungi form a large biodiversity and determines the chemical diversity of metabolites. There is a complex interaction between ticks and fungi. Ticks provide the necessary conditions for fungi to survive, while fungal metabolites serve as nutrients to ticks, nonetheless, some pathogenic fungi can also cause their death [22–25]. In this study, the community composition and diversity of the fungal microbiome in ticks were analyzed by high-throughput sequencing. The results provide a basis for understanding the interactions between ticks and fungi, thus helping to prevent and control tick-borne diseases.

## 2. Materials and Methods

### 2.1. Sample Collection and Preparation

We started collecting ticks from March to April 2022. Nonparasitic ticks were collected by flag-drag approach on the vegetation layer during the daytime, in addition, parasitic ticks were collected from sheep. All samples were shipped to the laboratory under dry ice conditions and then stored at −80°C. In the laboratory phase, wash ticks with absolute alcohol for 1 min to remove all pollutants, then rinse with ultrapure water for 3 min to remove absolute alcohol, and finally store it in the refrigerator (− 80°C) until morphological identification or nucleic acid extraction. We observed the appearance of *Ixodes* using a digital video microscope model HiROX MXB-2016Z and classified different ticks according to the different morphological characteristics [26]. Then mitochondrial genes 12S rRNA [27] and 16S rRNA [28] were used to further identify the species of ticks.

### 2.2. Nucleic Acid Extraction

After morphological identification, the collected ticks were separated into 50 groups based on their location, with each group consisting of pooled samples of 5 ticks per sample. Rinse nucleic acids with anhydrous ethanol before extracting them to remove surface stains and pathogens. Then put the tick into the centrifuge tube of 1.5 ml, add steel ball, RLT, and protease K, and centrifuge at 65-Hz 12,000*g* for 500 s at 4°C. After 200 *µ*l of supernatant, according to the manufacturer's instructions, use a DNA extraction kit (Tianlong, Xi'an, China) to extract nucleic acid. DNA concentration and integrity were measured by NanoDrop 2000c spectrophotometer (Thermo Fisher Scientific, Waltham, MA, USA) and agarose gel electrophoresis, respectively.

### 2.3. Molecular Identification of Tick by Polymerase Chain Reaction (PCR)

We selected representative samples for molecular characterization to further verify the results of the morphological classification of *Dermacentor nuttalli*, sequence typing was carried out by using DNA markers of the tick genome, including two mitochondrial genes 12S rRNA and 16S rRNA. The PCR primers for the genes are listed in Table 1, and the system and procedure of PCR are presented in Tables 2 and 3. The obtained nucleotide sequence was compared with those available in GenBank, and multiple sequence alignment was performed using the default parameters in MEGA11. Phylogenetic analysis was performed using MEGA11 using the maximum-likelihood (ML) method based on MEGA11 with an estimated Bootstrap value of 1,000 replicates.

### 2.4. Library Construction and DNA Sequencing

Internal transcribed spacer (ITS) regions 1 and 2 were amplified using two primers (forward, GCATCGATGAAGAACGCAGC; reverse, TCCTCCGCTTATTGATATGC). The primers were synthesized by Invitrogen (Invitrogen, Carlsbad, CA, USA). The reaction mixture contained 25 *μ*l of 2 × Premix Taq (TaKaRa Biotechnology, Dalian, China), 1 *μ*l of each primer (10 *μ*M), and 3 *μ*l of the DNA template (20 ng/*μ*l) in a total volume of 50 *µ*l. Amplification conditions consisted of a denaturation step at 94°C for 5 min, followed by 30 cycles at 94°C for 30 s, 52°C for 30 s, and 72°C for 30 s, and an elongation cycle at 72°C for 10 min. PCR was performed on an S1000 thermal cycler (Bio-Rad Laboratories, CA, USA). PCR products were analyzed by 1% agarose gel electrophoresis and quantified by a densitometry using GeneTools software version 4.03.05.0 (Syngene, Frederick, MD, USA). PCR products were purified using an EZNA Gel Extraction Kit (Omega Bio-Tek, GA, USA) and mixed into equimolar ratios.

DNA (2 *μ*l samples) was randomly fragmented and subjected to end-repair, A-tailing, and adaptor ligation. DNA concentration was quantified by fluorometry (Qubit 4.0). The integrity and size of DNA fragments were assessed using a high-throughput nucleic acid and protein analysis system (Qsep400; Houze Biological Technology Co., Hangzhou, China). Index codes were added to each sample, and coded samples were clustered on the CBOT cluster generation system according to the manufacturer's instructions. Sequencing was performed by the Guangdong Magigene Biotechnology (Guangzhou, China) on an Illumina NovaSeq 6000 high-throughput sequencing platform in PE250 mode [31, 32].

### 2.5. Bioinformatics Analysis

After obtaining the raw data, we preprocess the sequencing data: carry on the quality control statistics to the raw data according to the barcode sequence and the primer sequence, remove the barcode sequence and retain the front and back primer sequence [33].

Raw sequences were processed and assigned to amplicon sequence variants (ASVs) using the Divisive Amplicon Denoising Algorithm 2 (DADA-2) [34] (i.e., 100% operational taxonomic units (OTUs)) through the Quantitative Insights into Microbial Ecology 2 (QIIME2) [35] pipeline. OTUs were clustered at 97% sequence identity. Contig sequences were assembled using DNAMAN software version 6 (Lynnon Biosoft, Quebec, Canada). One ITS (internal transcribed spacer) sequence representative of each OTU was queried against GenBank using BLASTn. Mitochondrial and chloroplast sequences were removed. BLAST output files were extracted, entered into contingency tables, and converted into BIOM format.

### 2.6. Fungi Diversity and Taxonomic Analysis

Species accumulation curves were plotted using R version 4.2.2. Species richness (Chao1), Shannon index, and Simpson index were calculated using the vegan package in R. Principle coordinates analysis (PCoA) was performed using the ade4 package in R. Heatmaps were generated using the pheatmap package in R. Fungal taxa differentially abundant between the two tick groups were identified using the linear discriminant analysis (LDA) effect size (LEfSe) method (http://huttenhower.sph.harvard.edu/lefse/). Significant differences in the number of OTUs between the groups were analyzed using Statistical Analyses of Metagenomic Profiles (STAMP) software (http://kiwi.cs.dal.ca/Software/STAMP). According to the relative abundance of fungi in the samples, the top 42 fungal genera were analyzed to clarify the correlation among different fungal genera. The phylogenetic diversity of fungal genera was analyzed using the picante package in R with a correlation coefficient of >0.3. The phylogenetic networks were visualized using Gephi version 0.9.2.

### 2.7. Statistical Analysis

PCoA analysis based on Bray–Curtis dissimilarities was performed using permutational multivariate analysis of variance (PERMANOVA). Welch's *t*-test was used for group comparison analysis in STAMP. Co-occurrence network analysis was employed with spearman correlation value >0.3. The level of significance used in these analyses was 0.05.

## 3. Results

### 3.1. Tick Identification and Sequencing Data Statistics

**Figure 1:**
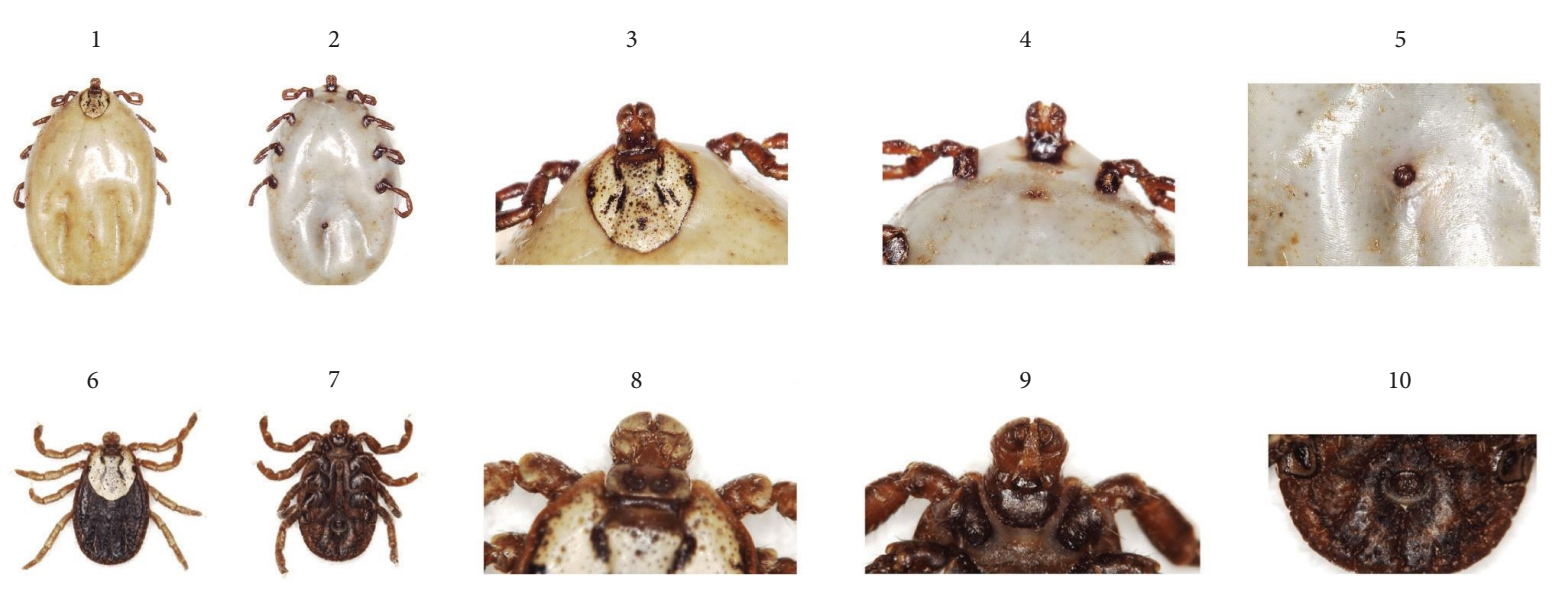
Dorsal and ventral views of adults of *Dermacentor nuttalli*. (1–5: parasitic ticks, 6–10: nonparasitic ticks).

**Figure 2:**
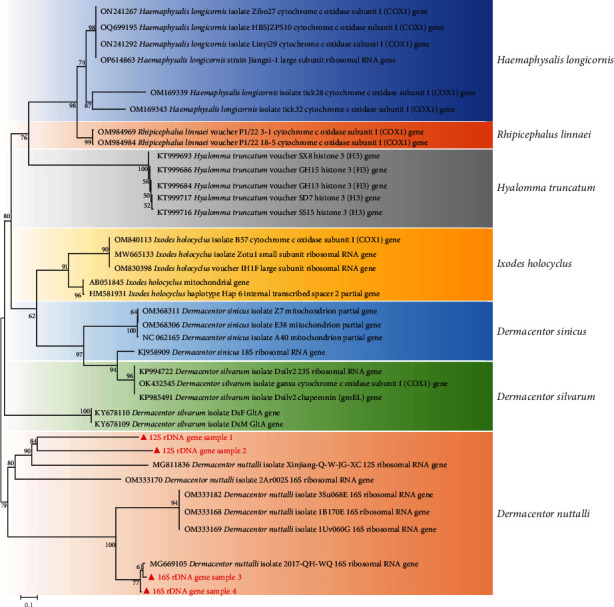
Phylogenetic tree based on 12S rRNA (a) and 16S rRNA (b) gene sequencing.

The tick species collected in the study area was *Dermacentor nuttalli* (Ixodidae) based on morphological analysis (Figure 1), species-specific PCR, sequence alignment, and phylogenetic analysis (Figure 2). A total of 7,482 ASVs were obtained. The sequencing coverage was close to 1, and the rank-abundance curve showed high and uniform species composition, indicating that the sequencing depth was sufficient to study the fungal microbiota. Secondary leveling was performed at the minimum sequencing depth to standardize the number of sequences in each sample.

### 3.2. Fungi Composition of *Dermacentor Nuttalli*

**Figure 3:**
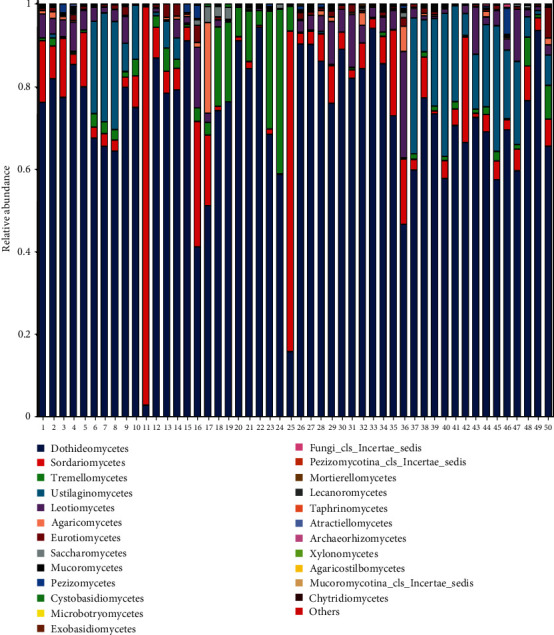
Relative abundance of fungal classes in *Dermacentor nuttalli* ticks collected from parasitic ticks (1–25) or nonparasitic ticks (26–50) in Wuwei, Gansu Province, China.

**Figure 4:**
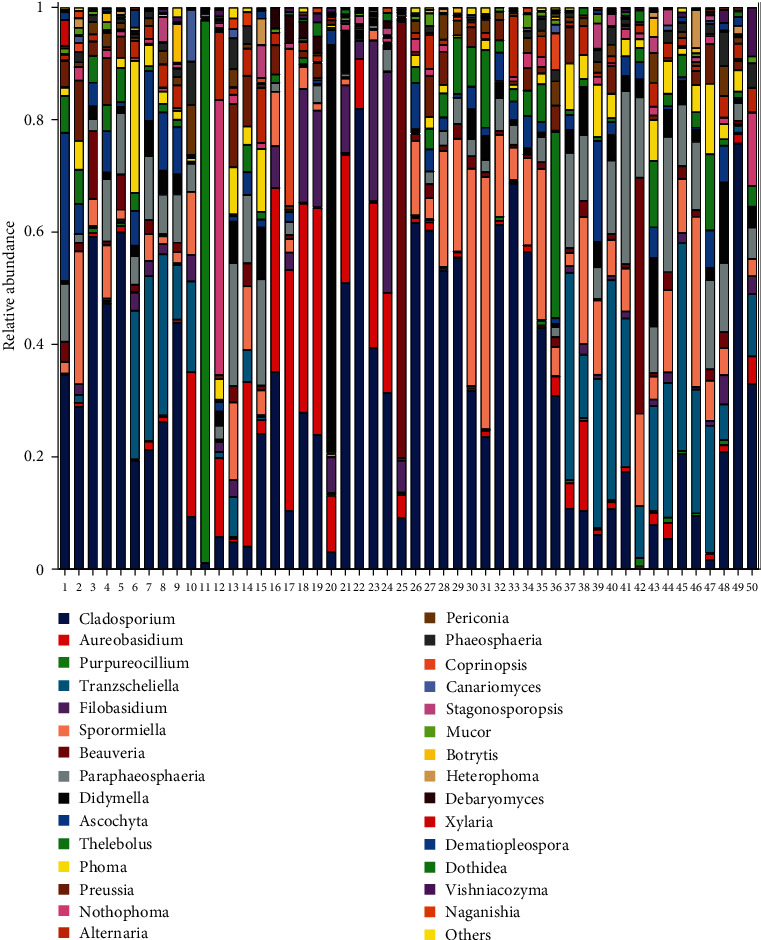
Relative abundance of the fungal genus in *Dermacentor nuttalli* ticks collected from parasitic ticks (1–25) or nonparasitic ticks (26–50) in Wuwei, Gansu Province, China.

Taxonomic classifications were performed using the UNITE database (https://unite.ut.ee/) [36]. The 7,482 ASVs were classified into four phyla, 18 classes, 46 orders, 116 families, 261 genera, and 385 species. The most abundant fungal classes were *Dothideomycetes*, *Sordariomycetes*, *Tremellomycetes*, *Leotiomycetes*, *Agaricomycetes*, and *Eurotiomycetes* (Figure 3). The most abundant genera in both tick groups were *Cladosporium*, *Aureobasidium*, *Purpureocillium*, and *Tranzscheliellla* (Figure 4).

### 3.3. Microbial Diversity

The dilution curve of each tick group was obtained to determine whether the sequencing depth was sufficient to meet the requirements of each sample. Although the rarefaction curves for Chao1 ASVs were close to saturation (*Supplementary 1*), the Shannon index and Simpson reached a stable value (*Supplementary 1*). Sequencing coverage in each group of samples varied between 95% and 100%, indicating that sequencing depth was sufficient to study the fungal microbiota. Secondary leveling was performed at the minimum sequencing depth to standardize the number of sequences in each sample.

**Figure 5:**
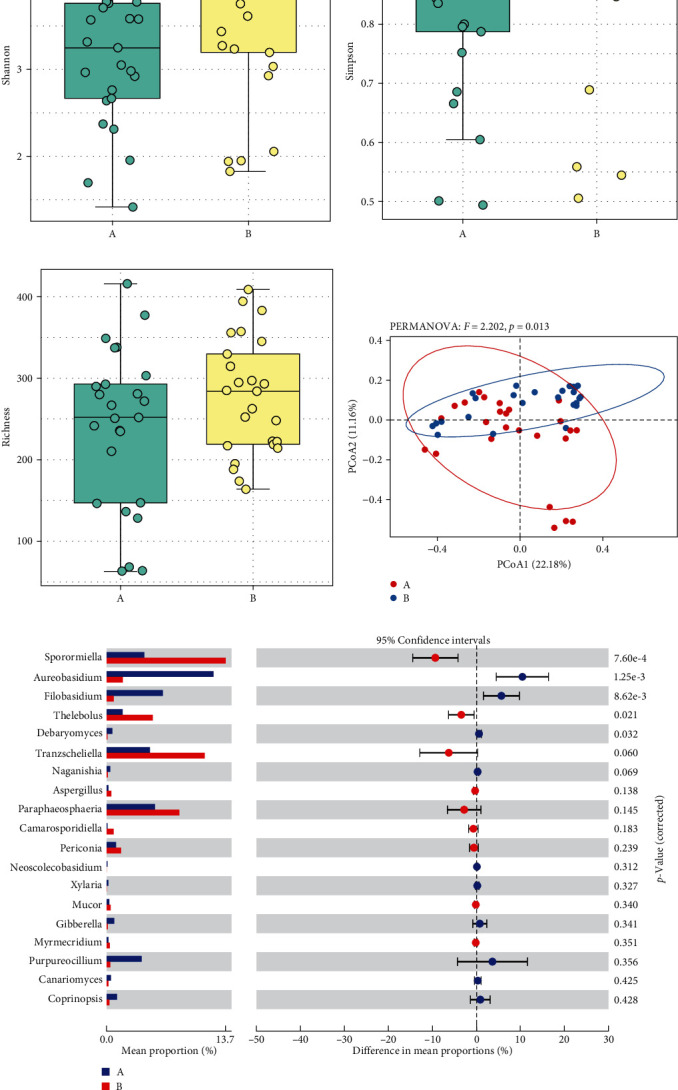
(a–c) Fungal diversity in *Dermacentor nuttalli* collected from parasitic ticks or nonparasitic ticks in Wuwei, Gansu Province, China, the dot points represent the index of each of the samples. (a) Boxplot showed fungi alpha diversity between two groups based on the Shannon index. (b) Boxplot showed fungi alpha diversity between two groups based on the Simpson index. (c) Boxplot showed fungi alpha diversity between two groups based on the richness index. (d) Beta diversity based on principal coordinate analysis of weighted UniFrac distances. (e) The extended error barplot shows the abundances of different fungi abundances in the two groups of samples. The middle shows the abundances of different species within the 95% confidence intervals. The value on the far right is the *p*-value. A (blue) and B (red) represent ticks collected from parasitic ticks and nonparasitic ticks, respectively.

**Figure 6:**
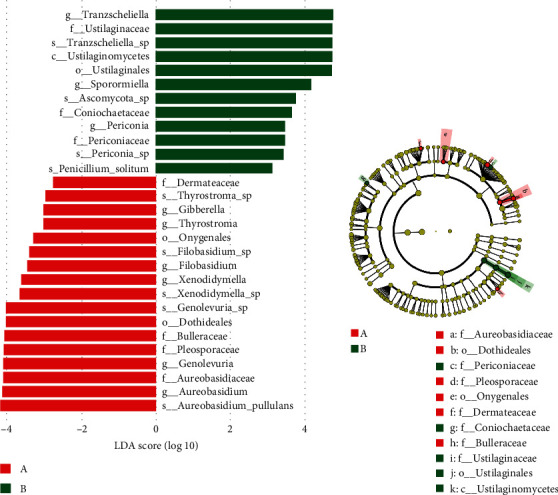
(a) Significantly enriched taxa (linear discriminant analysis score >2 and *p* < 0.05) in *Dermacentor nuttalli* collected from parasitic ticks or non-parasitic ticks in Wuwei, Gansu Province, China. (b) Cladogram showing the relationship between fungal taxa (phylum, class, order, family, and genus from inner to outer rings) in the tick groups.

There were no significant differences in Shannon, Simpson, and Chao1 indexes between the tick groups (*p* < 0.05) (Figure 5(a)–5(c)). The richness and evenness of fungal communities were similar between the groups. While cluster analysis did not show that ticks were significantly isolated from all samples, PCoA based on weighted UniFrac distances indicated significant clustering of fungal communities in the two groups at the ASV level, as determined by PERMANOVA (*F* = 2.202; *p*=0.013). In addition, PCoA1 and PCoA2 explained 22.18% and 11.16% of data variation, respectively (Figure 5(d)). To further explore the differential species carrying fungi between the two groups, they were analyzed for fungal abundance differences, and the results showed that there were more *Aureobasidium* and *Filobasidium* in parasitic ticks, and more Sporormiella and Tranzscheliella in nonparasitic ticks (Figure 5(e), *p* < 0.05). We also used LEfSe to identify differential microbial abundances between parasitic ticks and nonparasitic ticks (Figure 6). This analysis revealed significant differences (LDA ≥ 2, *p* < 0.05 determined by the Wilcoxon signed-rank test) in fungi clades from phylum to genus levels between the two groups.

### 3.4. Co-Occurrence Network Analysis

**Figure 7:**
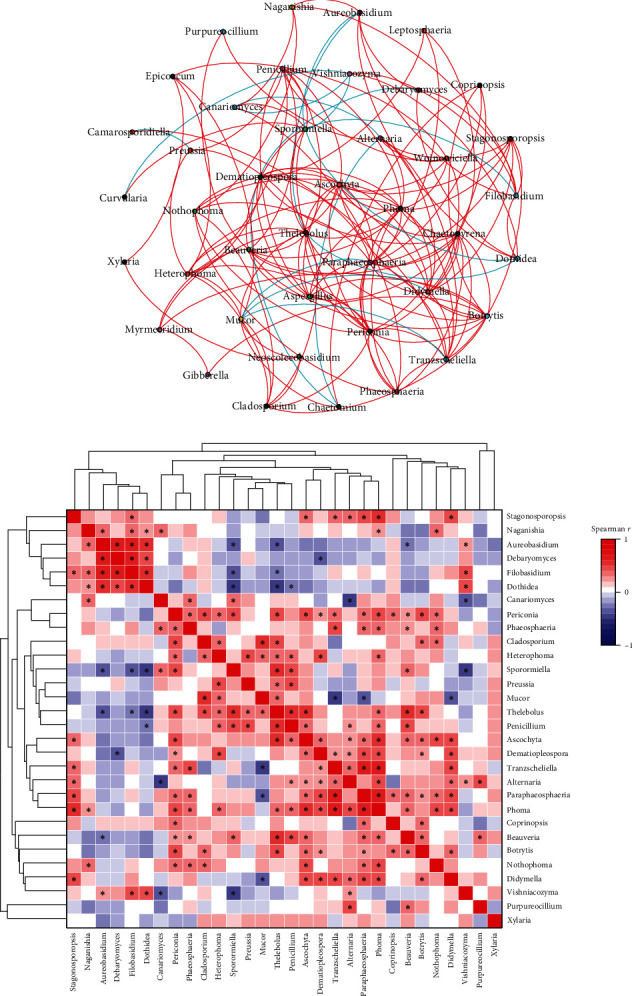
Co-occurrence network analysis and correlation heatmap of fungal genera. (a) Co-occurrence network analysis of fungal genera. Each node represents a genus, and red and blue lines represent positive and negative correlations, respectively (*p* < 0.05). (b) Heatmap of Spearman's correlations between the 30 most abundant genera  ^*∗*^*p* < 0.05.

To further explore the correlation between fungal microbial communities, co-occurrence network analysis with Spearman correlation was performed on fungal abundance (Figure 7(a); *Supplementary 2*). Co-occurrence network analysis showed the co-occurrence of several fungal genera, including *Xylaria* and *Neoscolecobasidium* (Spearman *r* = 0.714, *p* < 0.05), *Aureobasidium* and *Debaryomyces* (Spearman *r* = 0.700, *p* < 0.05), *Paraphaeosphaeria* and *Phoma* (Spearman *r* = 0.697, *p* < 0.05), *Tranzscheliella* and *Phoma* (Spearman *r* = 0.687, *p* < 0.05), *Sporormiella* and *Dothidea* (Spearman *r* = −0.456, *p* < 0.05), *Alternaria* and *Canariomyces* (Spearman *r* = −0.435, *p* < 0.05), and *Thelebolus* and *Dothidea* (Spearman *r* = −0.421, *p* < 0.05).

### 3.5. Interaction of Endosymbiont Fungi

Spearman's correlation coefficients (*r*) indicated that many bacterial genera were significantly correlated with each other (Figure 7(b); *Supplementary 1*). For instance, *Filobasidium* was positively correlated with *Aureobasidium* (Spearman *r* = 0.638, *p* < 0.05); *Paraphaeosphaeria* was positively correlated with *Tranzscheliella* (Spearman *r* = 0.614, *p* < 0.05); *Thelebolus* was positively correlated with *Beauveria* (Spearman *r* = 0.560, *p* < 0.05), and *Ascochyta* (Spearman *r* = 0.583, *p* < 0.05); *Phoma* was positively correlated with *Tranzscheliella* (Spearman *r* = 0.677, *p* < 0.05), and *Paraphaeosphaeria* (Spearman *r* = 0.702, *p* < 0.05); *Sporormiella* was negatively correlated with *Filobasidium* (Spearman *r* = −0.411, *p* < 0.05); *Thelebolus* was negatively correlated with *Filobasidium* (Spearman *r* = −0.394, *p* < 0.05). However, there was no significant relationship between reaction variables and explanatory variables.

## 4. Discussion

Ticks can transmit a wide range of pathogens, including bacteria, viruses, and fungi [37, 38]. In addition, the multihost life cycle facilitates pathogen transmission between cofeeding ticks [39, 40], when humans enter their life circle, they often become unexpected hosts, thus gaining the risk of infection [41]. At present, with the change in ecological conditions and human invasion of wildlife areas, the loss of biodiversity is intertwined with global climate change, which contributes to the current trend of the high incidence of tick-borne diseases in the world [42]. Tick-borne pathogenic (TBPs) diseases will continue to be prevalent among humans and animals in the foreseeable future, seriously endangering human and animal health [43].

The community composition and diversity of the fungal microbiota in *Dermacentor nuttalli* were assessed by high-throughput sequencing of the ITS gene. The most abundant fungal classes were *Dothideomycetes*, *Sordariomycetes*, *Tremellomycetes*, and *Leotiomycetes*. Renowned as one of the fungi closely related to plants, members of *Dothideomycetes* cause disease in crops [44]. The most abundant genera were *Cladosporium*, *Aureobasidium*, and *Purpureocillium*, and some species in these genera are opportunistic pathogens. For instance, *Cladophialophora bantiana* (formerly known as *Xylohypha bantiana*, *Cladosporium trichoides*, and *Cladophialophora bantianum*) is generally considered a neurotropic species, it can be isolated from living mammalian tissue, most commonly in patients with encephalitis [45]. These infections probably occur through inhalation and are fatal if untreated; overall survival is 28%–35%, and the most common clinical manifestations are lethargy, quadriplegia, and epilepsy [46, 47]. Additionally, studies on Nishimura and Miyaji [48] have suggested that the use of glucocorticoid can not only inhibit cellular immunity of the body but also promote the infection of *Cladophialophora bantianum*. Although the mechanism of infection is currently unclear, it is speculated from the clinical symptoms and indicators that blood-derived transmission may be the main mechanism [49]. Among all the fungal species of genera *Aureobasidium*, *A. pullulans* is a well-known species of pathogens that can cause human diseases [50]. *A. pullulans* is a saprophytic dematiaceous fungus that causes skin infections, meningitis, eye infections, and peritonitis, especially in immunosuppressed patients [51]. This species produces a pigment similar to melanin but is less pathogenic than other fungi. However, the pathogenesis of these infections is unclear [52–54]. *Purpureocillium lilacinum* (formerly known as *Paecilomyces lilacinus*), from the family Ophiocordycipitaceae, infects nematodes and causes eye infections in patients with chronic keratopathy, corneal injury, and a history of eye surgery [55–57]. *Purpureocillium lilacinum* causes mycotic keratitis and hyalohyphomycosis and has a poor prognosis, normally, it leads to endophthalmitis, and even the eyeball is removed in severe cases [58]. Although there are few reports on the pathogenic causes, one of the main causes of keratitis caused by *P. lilacinum* is its resistance to antifungal drugs [59].

There were marginal differences in fungal community richness and diversity between the tick groups. Nonetheless, PCoA of Bray–Curtis distances showed that fungal abundance differed significantly between the two groups (*p*=0.013). Similarly, the composition and diversity of fungal families differed across the groups, which might be attributed to the environmental factors, tick developmental stage, sex, and geographical area [27, 60, 61].

There were intergroup differences in the abundance of *Aureobasidium*, *Filobasidium*, *Sporormiella*, *Thelebolus*, and *Tranzscheliella*. *Aureobasidium* and *Filobasidium* were more prevalent in ticks infecting hosts, while *Sporormiella*, *Thelebolus*, and *Tranzscheliella* were more prevalent in ticks collected in the environment. Although *Purpureocillium* and *Aureobasidium* are opportunistic pathogens, their abundance was higher in parasitic ticks. This phenomenon may be due to changes in fungal abundance after hematophagy.

In the correlation analysis, we found that there were significant correlations between *Stagonosporopsis* and *Phoma*, *Cladosporium*, *Periconia*, and *Aureobasidium*, and between them and other genera. Just as some opportunistic pathogens mentioned above, although people are well aware of the conditions and mechanisms that lead to human diseases, little is known about the relationship between *Cladophialophora bantiana*, *Aureobasidium pullulans*, and ticks. It seems that many different fungal genera coexist with TBPs, and the symbiotic form can increase the colonization potential of pathogen forms. The pathogenicity of many fungi and the interaction between fungi and other microorganisms are not completely clear [20], which is also an important research direction of our work in the future. However, fungi and their hosts coexist at different stages and may play an active or negative role in the spread of pathogens [15, 62].

This study has limitations. First, the development and survival of microorganisms carried by ticks vary depending on tick sex and species and environmental factors [63, 64]. Second, the effects of fungal metabolites on the tick microbiome were not assessed. Thus, in future research, it should be important to control these factors in the analysis of mycobiome in *Dermacentor nuttalli*. In summary, the present study provides sufficient evidence that the mycobiome carried by ticks is rich and diverse, and that the differences between parasitic and nonparasitic ticks carrying pathogens are significant, it improved our knowledge about the mycobiome in ticks. Therefore, these findings can be used as the basis for future research to provide information and data for the development of prevention and control of TBPs.

## 5. Conclusion

In conclusion, this study demonstrated the mycobiome carried by *Dermacentor nuttalli* had rich diversity. There was a significant difference in mycobiome between parasitic ticks and nonparasitic ticks, and the parasitic ticks carried more opportunistic pathogens. Nonetheless, given the lack of research in the field of tick-borne fungi, this result provides help for the epidemiology of pathogens in a veterinary and public health sense. It also provides a new idea for further understanding the behavioral characteristics of ticks and establishing effective biological control measures.

## Figures and Tables

**Table 1:** Primers sequence information used in this study.

Target	Gene	Primer name	Sequence (5′-3′)	Reference
Tick	12S rDNA	T1B	AAACTAGGATTAGATACCCTATTATTTTAG	[29]
		T2A	CTATGTAACGACTTATCTTAATAAAGAGTG	
Tick	16S rDNA	16S-F	CTGCTCAATGATTTTTTAAATTGCTG	[30]
		16S- R	CCGGTCTGAACTCAGATCAAGT	
Microbiome	ITS 2-1	ITS-F	GCATCGATGAAGAACGCAGC	
		ITS-R	TCCTCCGCTTATTGATATGC	

**Table 2:** System of PCR in this study.

DreamTaq PCR master mix (2x)	25 *μ*l
Forward primer	2 *μ*l
Reverse primer	2 *μ*l
Template DNA	2 *μ*l
Water, nuclease-free	19 *μ*l
Total volume	50 *μ*l

**Table 3:** The procedure of PCR in this study.

Gene	Step	Temperature (°C)	Time	Cycles
16S rDNA	Initial denaturation	95	3 min	1
	Denaturation	95	30 s	35
	Annealing	50	30 s	
	Extension	72	1 min	
	Final extension	72	10 min	1

12S rDNA	Initial denaturation	95	3 min	1
	Denaturation	95	30 s	35
	Annealing	50	30 s	
	Extension	72	1 min	
	Final extension	72	10 min	1

## Data Availability

The sequencing data used to support the findings of this study are available from the corresponding author upon request.

## Supplementary Materials

Supplementary 1 Figure S1: Chao diversity indexes for group tick samples based on rarefaction curves of OTUs. Figure S2: Shannon diversity indexes for group tick samples based on rarefaction curves of OTUs. Figure S3: Simpson diversity indexes for group tick samples based on rarefaction curves of OTUs.

Supplementary 2 Table S1: results of co-occurrence network analysis of the fungal genus. Table S2: *P* values of Spearman correlation heatmap among 30 most abundant genera.
