# Supplementary material for: Revealing the Diversity of the Mycobiome in Different Phases of Ticks: ITS Gene-Based Analysis
Source: Transbound Emerg Dis. 2024 Jan 8;2024:8814592. doi: 10.1155/2024/8814592 (PMC12017015; doi:10.1155/2024/8814592)

Fig 1.Chao diversity indexes for group tick samples based on rarefaction curves of OTUs.

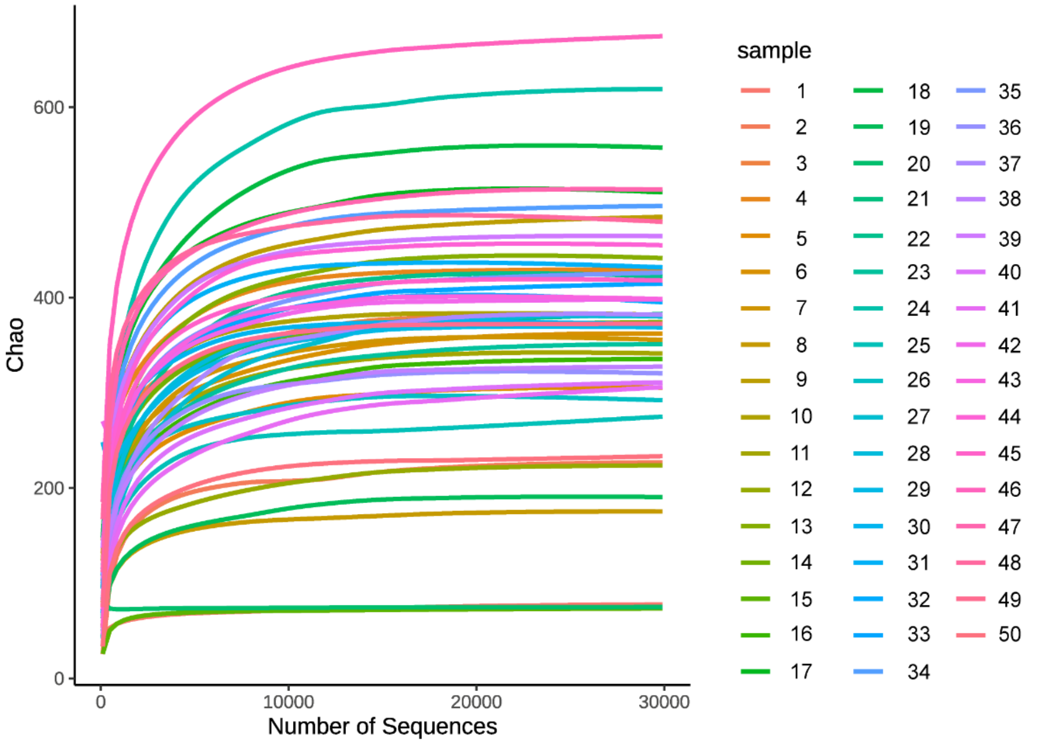

Fig 2. Shannon diversity indexes for group tick samples based on rarefaction curves of OTUs.

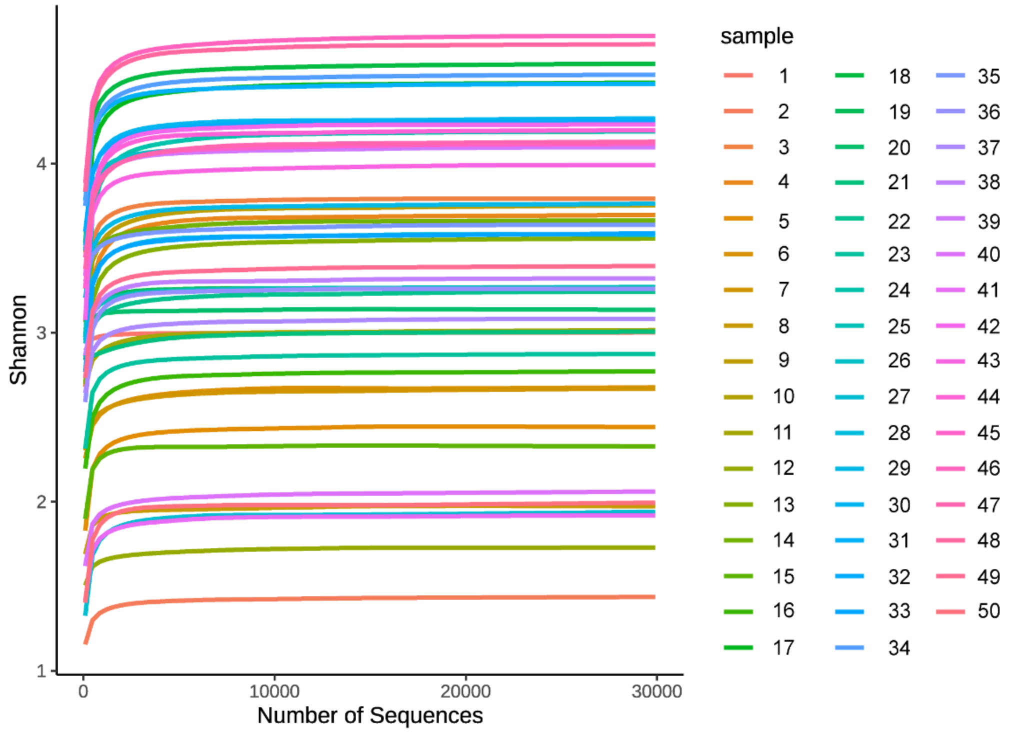

Fig 3. Simpson diversity indexes for group tick samples based on rarefaction curves of OTUs.

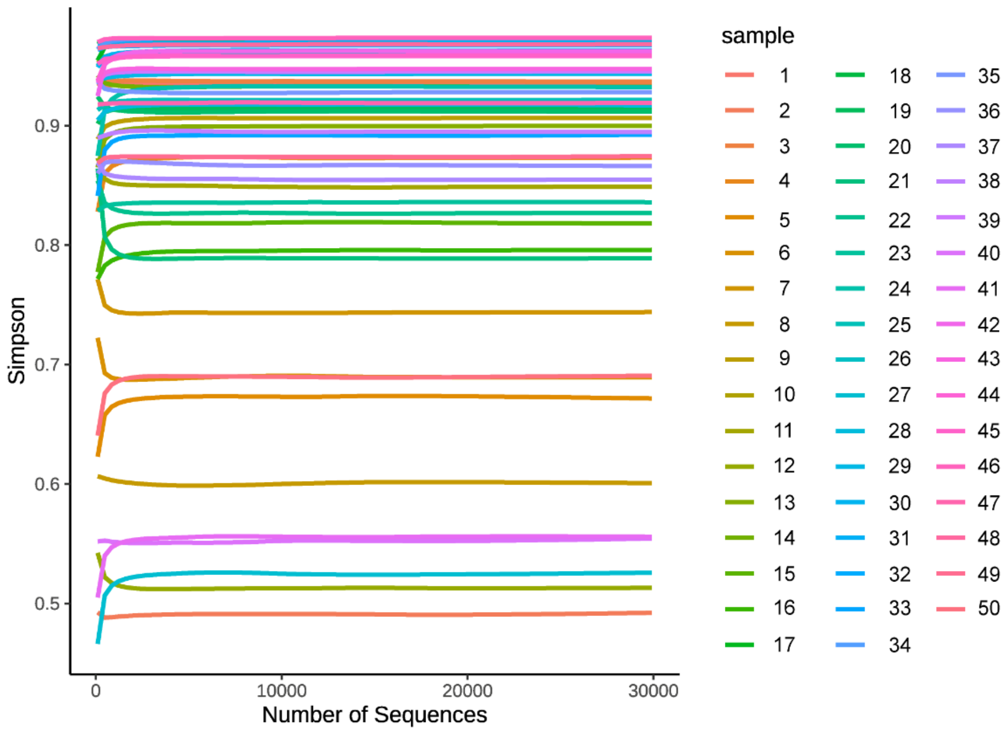

Supplement: Supplementary 1 — Figure S1: Chao diversity indexes for group tick samples based on rarefaction curves of OTUs. Figure S2: Shannon diversity indexes for group tick samples based on rarefaction curves of OTUs. Figure S3: Simpson diversity indexes for group tick samples based on rarefaction curves of OTUs. [file 8814592.f1.pdf]
