# Supplementary material for: Revealing the Diversity of the Mycobiome in Different Phases of Ticks: ITS Gene-Based Analysis
Source: Transbound Emerg Dis. 2024 Jan 8;2024:8814592. doi: 10.1155/2024/8814592 (PMC12017015; doi:10.1155/2024/8814592)
Supplement: Supplementary 2 — Table S1: results of co-occurrence network analysis of the fungal genus. Table S2: P values of Spearman correlation heatmap among 30 most abundant genera. [file 8814592.f2.pdf]

**Table S1. Results of co-occurrence network analysis of the fungal genus.**

| Target | Source | correlation | pvalue | po_ne |
|---|---|---|---|---|
| Alternaria | Chaetomium | -0.33211 | 0.01846 | negative |
| Alternaria | Stagonosporopsis | 0.302408 | 0.032801 | positive |
| Alternaria | Canariomyces | -0.43501 | 0.001594 | negative |
| Alternaria | Wojnowiciella | 0.398021 | 0.004202 | positive |
| Ascochyta | Thelebolus | 0.531057 | 7.26E-05 | positive |
| Ascochyta | Aspergillus | 0.351776 | 0.01224 | positive |
| Ascochyta | Phoma | 0.531689 | 7.09E-05 | positive |
| Ascochyta | Botrytis | 0.390145 | 0.005096 | positive |
| Ascochyta | Wojnowiciella | 0.307298 | 0.029947 | positive |
| Ascochyta | Periconia | 0.441491 | 0.00133 | positive |
| Ascochyta | Dematiopleospora | 0.366407 | 0.008869 | positive |
| Ascochyta | Penicillium | 0.388109 | 0.005352 | positive |
| Ascochyta | Chaetopyrena | 0.351354 | 0.012351 | positive |
| Ascochyta | Stagonosporopsis | 0.333666 | 0.017884 | positive |
| Ascochyta | Nothophoma | 0.466279 | 0.000643 | positive |
| Aspergillus | Chaetopyrena | 0.389466 | 0.00518 | positive |
| Aureobasidium | Filobasidium | 0.607642 | 2.87E-06 | positive |
| Aureobasidium | Debaryomyces | 0.699906 | 1.55E-08 | positive |
| Aureobasidium | Dothidea | 0.573553 | 1.34E-05 | positive |
| Aureobasidium | Sporormiella | -0.31938 | 0.02377 | negative |
| Aureobasidium | Thelebolus | -0.30257 | 0.032702 | negative |
| Aureobasidium | Naganishia | 0.313456 | 0.026651 | positive |
| Beauveria | Myrmecridium | 0.339159 | 0.015977 | positive |
| Beauveria | Ascochyta | 0.341094 | 0.015347 | positive |
| Beauveria | Paraphaeosphaeria | 0.342131 | 0.015019 | positive |
| Beauveria | Penicillium | 0.435211 | 0.001585 | positive |
| Beauveria | Phoma | 0.392747 | 0.004784 | positive |
| Beauveria | Thelebolus | 0.551202 | 3.35E-05 | positive |
| Beauveria | Botrytis | 0.322217 | 0.022487 | positive |
| Beauveria | Chaetopyrena | 0.397886 | 0.004216 | positive |
| Beauveria | Aspergillus | 0.330593 | 0.019034 | positive |
| Botrytis | Chaetopyrena | 0.495381 | 0.000255 | positive |
| Botrytis | Myrmecridium | 0.312414 | 0.027186 | positive |
| Botrytis | Gibberella | 0.359552 | 0.010332 | positive |
| Botrytis | Aspergillus | 0.370021 | 0.008172 | positive |
| Canariomyces | Vishniacozyma | -0.31573 | 0.025515 | negative |
| Canariomyces | Naganishia | 0.303409 | 0.032199 | positive |
| Chaetopyrena | Leptosphaeria | 0.403672 | 0.003649 | positive |
| Cladosporium | Heterophoma | 0.303462 | 0.032167 | positive |
| Cladosporium | Thelebolus | 0.440307 | 0.001375 | positive |
| Cladosporium | Neoscolecobasidium | 0.325431 | 0.021104 | positive |
| Cladosporium | Mucor | 0.57162 | 1.45E-05 | positive |
| Cladosporium | Nothophoma | 0.372206 | 0.007775 | positive |
| Cladosporium | Botrytis | 0.317067 | 0.024863 | positive |

| | | | | |
|---|---|---|---|---|
| Cladosporium | Periconia | 0.416095 | 0.002652 | positive |
| Coprinopsis | Chaetomium | 0.308526 | 0.029264 | positive |
| Coprinopsis | Botrytis | 0.315217 | 0.025766 | positive |
| Debaryomyces | Dothidea | 0.459783 | 0.000782 | positive |
| Debaryomyces | Dematiopleospora | -0.38954 | 0.005171 | negative |
| Dematiopleospora | Chaetomium | -0.30254 | 0.03272 | negative |
| Dematiopleospora | Leptosphaeria | 0.418697 | 0.002477 | positive |
| Dematiopleospora | Aspergillus | 0.360013 | 0.010228 | positive |
| Dematiopleospora | Chaetopyrena | 0.399798 | 0.004021 | positive |
| Didymella | Alternaria | 0.397913 | 0.004214 | positive |
| Didymella | Stagonosporopsis | 0.467237 | 0.000624 | positive |
| Didymella | Chaetopyrena | 0.410298 | 0.003083 | positive |
| Didymella | Mucor | -0.31057 | 0.028157 | negative |
| Didymella | Ascochyta | 0.400359 | 0.003965 | positive |
| Didymella | Dematiopleospora | 0.449115 | 0.001069 | positive |
| Didymella | Phoma | 0.499091 | 0.000225 | positive |
| Didymella | Botrytis | 0.309813 | 0.028562 | positive |
| Didymella | Wojnowiciella | 0.391202 | 0.004967 | positive |
| Dothidea | Vishniacozyma | 0.466419 | 0.00064 | positive |
| Filobasidium | Naganishia | 0.389445 | 0.005183 | positive |
| Filobasidium | Vishniacozyma | 0.474346 | 0.000501 | positive |
| Filobasidium | Stagonosporopsis | 0.320474 | 0.023269 | positive |
| Filobasidium | Dothidea | 0.540911 | 5.00E-05 | positive |
| Filobasidium | Debaryomyces | 0.647509 | 3.73E-07 | positive |
| Filobasidium | Sporormiella | -0.36094 | 0.01002 | negative |
| Gibberella | Myrmecridium | 0.324517 | 0.02149 | positive |
| Heterophoma | Epicoccum | 0.324502 | 0.021497 | positive |
| Heterophoma | Penicillium | 0.333241 | 0.01804 | positive |
| Heterophoma | Dematiopleospora | 0.411584 | 0.002982 | positive |
| Mucor | Neoscolecobasidium | 0.316124 | 0.025319 | positive |
| Mucor | Epicoccum | 0.409 | 0.003187 | positive |
| Mucor | Heterophoma | 0.317536 | 0.024638 | positive |
| Mucor | Chaetomium | 0.308378 | 0.029346 | positive |
| Nothophoma | Phaeosphaeria | 0.376544 | 0.007034 | positive |
| Nothophoma | Naganishia | 0.346678 | 0.013648 | positive |
| Nothophoma | Periconia | 0.306534 | 0.030379 | positive |
| Paraphaeosphaeria | Ascochyta | 0.338641 | 0.016149 | positive |
| Paraphaeosphaeria | Periconia | 0.357618 | 0.010781 | positive |
| Paraphaeosphaeria | Didymella | 0.479206 | 0.00043 | positive |
| Paraphaeosphaeria | Mucor | -0.30356 | 0.03211 | negative |
| Paraphaeosphaeria | Aspergillus | 0.311606 | 0.027607 | positive |
| Paraphaeosphaeria | Phaeosphaeria | 0.396637 | 0.004349 | positive |
| Paraphaeosphaeria | Chaetopyrena | 0.467494 | 0.000619 | positive |
| Paraphaeosphaeria | Stagonosporopsis | 0.454841 | 0.000905 | positive |
| Paraphaeosphaeria | Leptosphaeria | 0.305997 | 0.030685 | positive |
| Paraphaeosphaeria | Phoma | 0.697289 | 1.84E-08 | positive |
| Paraphaeosphaeria | Coprinopsis | 0.373017 | 0.007631 | positive |

| | | | | |
|---|---|---|---|---|
| Paraphaeosphaeria | Nothophoma | 0.338946 | 0.016047 | positive |
| Paraphaeosphaeria | Dematiopleospora | 0.404919 | 0.003536 | positive |
| Penicillium | Aspergillus | 0.303135 | 0.032362 | positive |
| Penicillium | Wojnowiciella | 0.558171 | 2.53E-05 | positive |
| Periconia | Aspergillus | 0.496411 | 0.000246 | positive |
| Periconia | Coprinopsis | 0.37101 | 0.00799 | positive |
| Periconia | Heterophoma | 0.317888 | 0.02447 | positive |
| Periconia | Botrytis | 0.491929 | 0.000285 | positive |
| Periconia | Chaetopyrena | 0.340285 | 0.015608 | positive |
| Periconia | Phaeosphaeria | 0.392149 | 0.004854 | positive |
| Phaeosphaeria | Chaetopyrena | 0.356977 | 0.010933 | positive |
| Phaeosphaeria | Aspergillus | 0.334369 | 0.01763 | positive |
| Phaeosphaeria | Canariomyces | 0.384995 | 0.005767 | positive |
| Phoma | Nothophoma | 0.413923 | 0.002807 | positive |
| Phoma | Stagonosporopsis | 0.530574 | 7.39E-05 | positive |
| Phoma | Heterophoma | 0.389247 | 0.005208 | positive |
| Phoma | Aspergillus | 0.405775 | 0.00346 | positive |
| Phoma | Chaetopyrena | 0.356624 | 0.011018 | positive |
| Phoma | Alternaria | 0.458478 | 0.000813 | positive |
| Phoma | Phaeosphaeria | 0.36207 | 0.009772 | positive |
| Phoma | Dematiopleospora | 0.461407 | 0.000745 | positive |
| Phoma | Periconia | 0.403939 | 0.003624 | positive |
| Phoma | Penicillium | 0.38311 | 0.006031 | positive |
| Phoma | Wojnowiciella | 0.502989 | 0.000197 | positive |
| Preussia | Camarosporidiella | -0.31405 | 0.026347 | negative |
| Preussia | Penicillium | 0.475893 | 0.000478 | positive |
| Preussia | Heterophoma | 0.441537 | 0.001328 | positive |
| Purpureocillium | Beauveria | 0.369284 | 0.00831 | positive |
| Purpureocillium | Alternaria | 0.406168 | 0.003426 | positive |
| Sporormiella | Vishniacozyma | -0.41632 | 0.002637 | negative |
| Sporormiella | Curvularia | 0.312025 | 0.027388 | positive |
| Sporormiella | Periconia | 0.422092 | 0.002264 | positive |
| Sporormiella | Penicillium | 0.433578 | 0.001658 | positive |
| Sporormiella | Beauveria | 0.348293 | 0.013188 | positive |
| Sporormiella | Dothidea | -0.45634 | 0.000866 | negative |
| Sporormiella | Thelebolus | 0.524349 | 9.29E-05 | positive |
| Sporormiella | Canariomyces | 0.371117 | 0.007971 | positive |
| Thelebolus | Penicillium | 0.566873 | 1.77E-05 | positive |
| Thelebolus | Preussia | 0.382545 | 0.006112 | positive |
| Thelebolus | Phoma | 0.34911 | 0.01296 | positive |
| Thelebolus | Aspergillus | 0.394721 | 0.004558 | positive |
| Thelebolus | Dothidea | -0.42069 | 0.00235 | negative |
| Thelebolus | Mucor | 0.3802 | 0.006459 | positive |
| Thelebolus | Botrytis | 0.477465 | 0.000455 | positive |
| Thelebolus | Heterophoma | 0.39353 | 0.004693 | positive |
| Thelebolus | Periconia | 0.448788 | 0.00108 | positive |
| Thelebolus | Myrmecridium | 0.520473 | 0.000107 | positive |

| | | | | |
|---|---|---|---|---|
| Tranzscheliella | Paraphaeosphaeria | 0.656203 | 2.30E-07 | positive |
| Tranzscheliella | Didymella | 0.406967 | 0.003357 | positive |
| Tranzscheliella | Chaetopyrena | 0.345863 | 0.013885 | positive |
| Tranzscheliella | Periconia | 0.325273 | 0.021171 | positive |
| Tranzscheliella | Phaeosphaeria | 0.437947 | 0.001469 | positive |
| Tranzscheliella | Alternaria | 0.374346 | 0.007401 | positive |
| Tranzscheliella | Camarosporidiella | 0.423021 | 0.002209 | positive |
| Tranzscheliella | Stagonosporopsis | 0.369579 | 0.008255 | positive |
| Tranzscheliella | Mucor | -0.40302 | 0.00371 | negative |
| Tranzscheliella | Phoma | 0.687585 | 3.47E-08 | positive |
| Vishniacozyma | Curvularia | -0.30914 | 0.028928 | negative |
| Wojnowiciella | Epicoccum | 0.319781 | 0.023586 | positive |
| Xylaria | Neoscolecobasidium | 0.714286 | 5.72E-09 | positive |
| Xylaria | Epicoccum | 0.402007 | 0.003805 | positive |

**Table S2. P values of Spearman correlation heatmap among 30 most abundant genera.**

| Label | Stagonosporopsis | Naganishia | Aureobasidium | Debaryomyces | Filobasidium | Dothidea | Canariomyces | Periconia |
|---|---|---|---|---|---|---|---|---|
| Stagonospo | -- | 0.20649 | 0.18495 | 0.27518 | 0.32047 | 0.2044 | 0.07339 | 0.05625 |
| Naganishia | 0.20649 | -- | 0.31346 | 0.1323 | 0.38944 | 0.28141 | 0.30341 | 0.15234 |
| Aureobasic | 0.18495 | 0.31346 | -- | 0.69991 | 0.60764 | 0.57355 | 0.03682 | -0.09483 |
| Debaryomy | 0.27518 | 0.1323 | 0.69991 | -- | 0.64751 | 0.45978 | 0.05032 | -0.19608 |
| Filobasidiu | 0.32047 | 0.38944 | 0.60764 | 0.64751 | -- | 0.54091 | -0.01262 | -0.07508 |
| Dothidea | 0.2044 | 0.28141 | 0.57355 | 0.45978 | 0.54091 | -- | -0.08317 | -0.22165 |
| Canariomy | 0.07339 | 0.30341 | 0.03682 | 0.05032 | -0.01262 | -0.08317 | -- | 0.19467 |
| Periconia | 0.05625 | 0.15234 | -0.09483 | -0.19608 | -0.07508 | -0.22165 | 0.19467 | -- |
| Phaeospha | 0.09332 | 0.22832 | 0.11642 | 0.0848 | 0.25102 | -0.07291 | 0.38499 | 0.39215 |
| Cladosporiu | 0.05449 | 0.0889 | 0.11843 | 0.18654 | 0.16068 | 0.17271 | -0.01838 | 0.4161 |
| Heteropho | 0.14501 | 0.05521 | -0.04708 | -0.13965 | -0.05424 | -0.07114 | 0.03053 | 0.31789 |
| Sporormiel | -0.165 | -0.12639 | -0.31938 | -0.14573 | -0.36094 | -0.45634 | 0.37112 | 0.42209 |
| Preussia | -0.05647 | 0.09937 | 0.03571 | 0.10202 | -0.02991 | -0.13233 | 0.26626 | 0.19787 |
| Mucor | -0.20622 | -0.07167 | -0.16357 | -0.06003 | -0.15551 | -0.13197 | 0.05075 | 0.08327 |
| Thelebolus | 0.03269 | -0.07903 | -0.30257 | -0.16377 | -0.29011 | -0.42069 | 0.12576 | 0.44879 |
| Penicillium | -0.01508 | -0.05522 | -0.18616 | -0.12048 | -0.19271 | -0.29585 | 0.10531 | 0.26192 |
| Ascochyta | 0.33367 | 0.13943 | -0.27328 | -0.14349 | -0.10487 | -0.23699 | 0.09197 | 0.44149 |
| Dematiople | 0.19383 | 0.07339 | -0.23258 | -0.38954 | -0.01633 | -0.15532 | -0.14031 | 0.2853 |
| Tranzscheli | 0.36958 | 0.16602 | -0.1347 | -0.17405 | 0.19324 | -0.06414 | 0.01685 | 0.32527 |
| Alternaria | 0.30241 | 0.00848 | -0.11148 | -0.14336 | 0.07552 | -0.03586 | -0.43501 | 0.11741 |
| Paraphaeo | 0.45484 | 0.14447 | -0.11777 | -0.05843 | 0.19619 | -0.02449 | 0.25241 | 0.35762 |
| Phoma | 0.53057 | 0.28017 | -0.08892 | -0.19316 | 0.08874 | -0.12465 | 0.13203 | 0.40394 |
| Coprinopsi | 0.22164 | -0.01697 | 0.14553 | 0.11033 | 0.11059 | 0.13922 | 0.21829 | 0.37101 |
| Beauveria | -0.07889 | -0.23672 | -0.29152 | -0.16838 | -0.16899 | -0.20683 | -0.04738 | 0.29073 |
| Botrytis | 0.08062 | -0.20626 | -0.18977 | -0.07369 | -0.1316 | -0.21515 | -0.09034 | 0.49193 |
| Nothophor | 0.24356 | 0.34668 | -0.00298 | -0.03577 | 0.19509 | 0.05763 | 0.26519 | 0.30653 |
| Didymella | 0.46724 | 0.13372 | -0.02007 | -0.02153 | 0.20552 | 0.02101 | -0.07315 | 0.1926 |
| Vishniacozy | 0.14291 | 0.03855 | 0.28607 | 0.24934 | 0.47435 | 0.46642 | -0.31573 | -0.16509 |
| Purpureoci | -0.00324 | -0.23639 | -0.16808 | -0.12801 | 0.03447 | 0.00293 | -0.27586 | 0.06206 |
| Xylaria | 0.09274 | 0.05356 | -0.21811 | -0.11201 | 0.02485 | -0.11608 | -0.09168 | -0.12443 |

| Phaeosphaeria | Cladosporium | Heterophoma | Sporormiella | Preussia | Mucor | Thelebolus | Penicillium | Ascochyta |
|---|---|---|---|---|---|---|---|---|
| 0.09332 | 0.05449 | 0.14501 | -0.165 | -0.05647 | -0.20622 | 0.03269 | -0.01508 | 0.33367 |
| 0.22832 | 0.0889 | 0.05521 | -0.12639 | 0.09937 | -0.07167 | -0.07903 | -0.05522 | 0.13943 |
| 0.11642 | 0.11843 | -0.04708 | -0.31938 | 0.03571 | -0.16357 | -0.30257 | -0.18616 | -0.27328 |
| 0.0848 | 0.18654 | -0.13965 | -0.14573 | 0.10202 | -0.06003 | -0.16377 | -0.12048 | -0.14349 |
| 0.25102 | 0.16068 | -0.05424 | -0.36094 | -0.02991 | -0.15551 | -0.29011 | -0.19271 | -0.10487 |
| -0.07291 | 0.17271 | -0.07114 | -0.45634 | -0.13233 | -0.13197 | -0.42069 | -0.29585 | -0.23699 |
| 0.38499 | -0.01838 | 0.03053 | 0.37112 | 0.26626 | 0.05075 | 0.12576 | 0.10531 | 0.09197 |
| 0.39215 | 0.4161 | 0.31789 | 0.42209 | 0.19787 | 0.08327 | 0.44879 | 0.26192 | 0.44149 |
| -- | 0.14692 | -0.15964 | 0.16952 | 0.05393 | 0.02783 | 0.14665 | 0.18924 | 0.2643 |
| 0.14692 | -- | 0.30346 | 0.06691 | 0.12175 | 0.57162 | 0.44031 | 0.13123 | 0.27222 |
| -0.15964 | 0.30346 | -- | 0.16434 | 0.44154 | 0.31754 | 0.39353 | 0.33324 | 0.17097 |
| 0.16952 | 0.06691 | 0.16434 | -- | 0.21126 | 0.15006 | 0.52435 | 0.43358 | 0.25834 |
| 0.05393 | 0.12175 | 0.44154 | 0.21126 | -- | 0.19255 | 0.38254 | 0.47589 | 0.21241 |
| 0.02783 | 0.57162 | 0.31754 | 0.15006 | 0.19255 | -- | 0.3802 | 0.13662 | 0.20034 |
| 0.14665 | 0.44031 | 0.39353 | 0.52435 | 0.38254 | 0.3802 | -- | 0.56687 | 0.53106 |
| 0.18924 | 0.13123 | 0.33324 | 0.43358 | 0.47589 | 0.13662 | 0.56687 | -- | 0.38811 |
| 0.2643 | 0.27222 | 0.17097 | 0.25834 | 0.21241 | 0.20034 | 0.53106 | 0.38811 | -- |
| 0.02868 | 0.13271 | 0.41158 | -0.07773 | -0.03904 | 0.03668 | 0.27397 | 0.11375 | 0.36641 |
| 0.43795 | -0.27136 | -0.0011 | 0.1613 | -0.14153 | -0.40302 | 0.0448 | 0.07013 | 0.255 |
| 0.07693 | 0.06767 | 0.14598 | -0.15173 | -0.1049 | -0.16675 | 0.20766 | 0.28601 | 0.28887 |
| 0.39664 | 0.06291 | 0.18168 | 0.19281 | 0.1216 | -0.30356 | 0.21169 | 0.19176 | 0.33864 |
| 0.36207 | 0.05632 | 0.38925 | 0.22943 | 0.20169 | -0.08183 | 0.34911 | 0.38311 | 0.53169 |
| 0.22677 | 0.27324 | 0.17037 | 0.11688 | -0.02561 | 0.03549 | 0.2193 | 0.01304 | 0.11106 |
| 0.29021 | 0.19637 | 0.27326 | 0.34829 | 0.16542 | 0.13585 | 0.5512 | 0.43521 | 0.34109 |
| 0.19908 | 0.31707 | 0.10845 | 0.20193 | -0.02514 | 0.22704 | 0.47747 | 0.19209 | 0.39015 |
| 0.37654 | 0.37221 | 0.22722 | 0.01899 | 0.07764 | 0.23919 | 0.18257 | 0.13159 | 0.46628 |
| 0.26807 | -0.0253 | -0.03656 | -0.0566 | -0.1876 | -0.31057 | 0.04072 | 0.17154 | 0.40036 |
| 0.027 | 0.12437 | 0.02467 | -0.41632 | -0.13394 | -0.06558 | -0.15092 | -0.07342 | 0.02134 |
| 0.11297 | -0.04585 | 0.01424 | -0.08717 | -0.04024 | -0.09611 | 0.07786 | 0.03821 | 0.03892 |
| -0.18507 | 0.23265 | 0.26316 | 0.13368 | 0.23485 | 0.24704 | 0.2335 | 0.23379 | 0.24286 |

| Dematiopleospora | Tranzscheliella | Alternaria | Paraphaeosphaeria | Phoma | Coprinopsis | Beauveria | Botrytis | Nothophoma |
|---|---|---|---|---|---|---|---|---|
| 0.19383 | 0.36958 | 0.30241 | 0.45484 | 0.53057 | 0.22164 | -0.07889 | 0.08062 | 0.24356 |
| 0.07339 | 0.16602 | 0.00848 | 0.14447 | 0.28017 | -0.01697 | -0.23672 | -0.20626 | 0.34668 |
| -0.23258 | -0.1347 | -0.11148 | -0.11777 | -0.08892 | 0.14553 | -0.29152 | -0.18977 | -0.00298 |
| -0.38954 | -0.17405 | -0.14336 | -0.05843 | -0.19316 | 0.11033 | -0.16838 | -0.07369 | -0.03577 |
| -0.01633 | 0.19324 | 0.07552 | 0.19619 | 0.08874 | 0.11059 | -0.16899 | -0.1316 | 0.19509 |
| -0.15532 | -0.06414 | -0.03586 | -0.02449 | -0.12465 | 0.13922 | -0.20683 | -0.21515 | 0.05763 |
| -0.14031 | 0.01685 | -0.43501 | 0.25241 | 0.13203 | 0.21829 | -0.04738 | -0.09034 | 0.26519 |
| 0.2853 | 0.32527 | 0.11741 | 0.35762 | 0.40394 | 0.37101 | 0.29073 | 0.49193 | 0.30653 |
| 0.02868 | 0.43795 | 0.07693 | 0.39664 | 0.36207 | 0.22677 | 0.29021 | 0.19908 | 0.37654 |
| 0.13271 | -0.27136 | 0.06767 | 0.06291 | 0.05632 | 0.27324 | 0.19637 | 0.31707 | 0.37221 |
| 0.41158 | -0.0011 | 0.14598 | 0.18168 | 0.38925 | 0.17037 | 0.27326 | 0.10845 | 0.22722 |
| -0.07773 | 0.1613 | -0.15173 | 0.19281 | 0.22943 | 0.11688 | 0.34829 | 0.20193 | 0.01899 |
| -0.03904 | -0.14153 | -0.1049 | 0.1216 | 0.20169 | -0.02561 | 0.16542 | -0.02514 | 0.07764 |
| 0.03668 | -0.40302 | -0.16675 | -0.30356 | -0.08183 | 0.03549 | 0.13585 | 0.22704 | 0.23919 |
| 0.27397 | 0.0448 | 0.20766 | 0.21169 | 0.34911 | 0.2193 | 0.5512 | 0.47747 | 0.18257 |
| 0.11375 | 0.07013 | 0.28601 | 0.19176 | 0.38311 | 0.01304 | 0.43521 | 0.19209 | 0.13159 |
| 0.36641 | 0.255 | 0.28887 | 0.33864 | 0.53169 | 0.11106 | 0.34109 | 0.39015 | 0.46628 |
| -- | 0.2796 | 0.2864 | 0.40492 | 0.46141 | 0.11716 | 0.26501 | 0.28031 | 0.07457 |
| 0.2796 | -- | 0.37435 | 0.6562 | 0.68758 | 0.05911 | 0.19613 | 0.14845 | 0.26398 |
| 0.2864 | 0.37435 | -- | 0.24708 | 0.45848 | -0.21882 | 0.2696 | 0.26435 | 0.20729 |
| 0.40492 | 0.6562 | 0.24708 | -- | 0.69729 | 0.37302 | 0.34213 | 0.29852 | 0.33895 |
| 0.46141 | 0.68758 | 0.45848 | 0.69729 | -- | 0.10718 | 0.39275 | 0.21294 | 0.41392 |
| 0.11716 | 0.05911 | -0.21882 | 0.37302 | 0.10718 | -- | 0.17632 | 0.31522 | 0.00386 |
| 0.26501 | 0.19613 | 0.2696 | 0.34213 | 0.39275 | 0.17632 | -- | 0.32222 | 0.03162 |
| 0.28031 | 0.14845 | 0.26435 | 0.29852 | 0.21294 | 0.31522 | 0.32222 | -- | 0.16279 |
| 0.07457 | 0.26398 | 0.20729 | 0.33895 | 0.41392 | 0.00386 | 0.03162 | 0.16279 | -- |
| 0.44911 | 0.40697 | 0.39791 | 0.47921 | 0.49909 | 0.04477 | 0.10133 | 0.30981 | 0.24325 |
| 0.18213 | 0.01888 | 0.29728 | -0.03899 | -0.11373 | -0.08824 | -0.02622 | -0.0683 | 0.04914 |
| 0.10436 | 0.16568 | 0.40617 | 0.04238 | 0.14526 | -0.23277 | 0.36928 | 0.21953 | 0.03407 |
| 0.2707 | -0.08011 | 0.18387 | 0.24263 | 0.19441 | -0.09187 | 0.23321 | -0.09976 | 0.03015 |

| Didymella | Vishniacozyma | Purpureocillium | Xylaria |
|---|---|---|---|
| 0.46724 | 0.14291 | -0.00324 | 0.09274 |
| 0.13372 | 0.03855 | -0.23639 | 0.05356 |
| -0.02007 | 0.28607 | -0.16808 | -0.21811 |
| -0.02153 | 0.24934 | -0.12801 | -0.11201 |
| 0.20552 | 0.47435 | 0.03447 | 0.02485 |
| 0.02101 | 0.46642 | 0.00293 | -0.11608 |
| -0.07315 | -0.31573 | -0.27586 | -0.09168 |
| 0.1926 | -0.16509 | 0.06206 | -0.12443 |
| 0.26807 | 0.027 | 0.11297 | -0.18507 |
| -0.0253 | 0.12437 | -0.04585 | 0.23265 |
| -0.03656 | 0.02467 | 0.01424 | 0.26316 |
| -0.0566 | -0.41632 | -0.08717 | 0.13368 |
| -0.1876 | -0.13394 | -0.04024 | 0.23485 |
| -0.31057 | -0.06558 | -0.09611 | 0.24704 |
| 0.04072 | -0.15092 | 0.07786 | 0.2335 |
| 0.17154 | -0.07342 | 0.03821 | 0.23379 |
| 0.40036 | 0.02134 | 0.03892 | 0.24286 |
| 0.44911 | 0.18213 | 0.10436 | 0.2707 |
| 0.40697 | 0.01888 | 0.16568 | -0.08011 |
| 0.39791 | 0.29728 | 0.40617 | 0.18387 |
| 0.47921 | -0.03899 | 0.04238 | 0.24263 |
| 0.49909 | -0.11373 | 0.14526 | 0.19441 |
| 0.04477 | -0.08824 | -0.23277 | -0.09187 |
| 0.10133 | -0.02622 | 0.36928 | 0.23321 |
| 0.30981 | -0.0683 | 0.21953 | -0.09976 |
| 0.24325 | 0.04914 | 0.03407 | 0.03015 |
| -- | 0.18213 | 0.24284 | 0.06961 |
| 0.18213 | -- | 0.08132 | 0.2282 |
| 0.24284 | 0.08132 | -- | -0.16823 |
| 0.06961 | 0.2282 | -0.16823 | -- |
